# Medication adherence in the treatment of depression

**DOI:** 10.1192/j.eurpsy.2023.392

**Published:** 2023-07-19

**Authors:** B. Pejuskovic, M. Lero, V. Pavlovic, G. Nikolasevic, V. Pesic

**Affiliations:** ^1^ Institute of Mental Health; ^2^Faculty of Medicine; ^3^Faculty of Pharmacy, Belgrade, Serbia

## Abstract

**Introduction:**

Depression is predicted to become one of the major sources of disease burden worldwide, leading to numerous adverse consequences that complicate the daily rhythm of life. Non-adherence is a serious issue in patients suffering from depression. Premature discontinuation of treatment is repeatedly encountered in depression, bringing on to increased disease severity, greater number of relapses, more hospitalizations and decreased remission rates. Given the impact of medication non-adherence among patients with depressive disorders, it is important to recognize factors associated with non-adherence and find ways to influence them.

**Objectives:**

Our objective was to find out the frequency, as well as potential differences in self-reported psychological distress of medical adherence in patients diagnosed with major depressive disorder.

**Methods:**

Sample consisted 83 patients (*M*_age_ = 45.4, *SD* = 14.8, 76% were female, 24% were male) with major depressive disorder (MDD) hospitalized at the Clinical Department of Crisis and Affective Disorders. After the informed consent of patients, the following assessment tools were administered: A socio-demographic questionnaire, Mini International Neuropsychiatric Interview (M.I.N.I.-6), Depression Anxiety Stress Scales (DASS-21), and The Morisky Medication Adherence Scale (MMAS-8).

**Results:**

Thirty-three (39.8%) patients were considered non-adherent (MMAS-8 adherence score < 6) while 45 (54.2%) had moderate adherence (MMAS-8 adherence score < 8) and 5 (6%) high adherence (MMAS-8 adherence score = 8) to their medication respectively. Negative associations were found between medication adherence and self-reported levels of depression (*r* = -0.30, *p* < 0.01), anxiety (*r* = -0.29. *p* < 0.01) and stress (*r* = -0.31, *p* < 0.01). One-way ANOVA yielded significant variation on the self-reported anxiety subscale of the DASS-21 questionnaire among adherence groups of patients with MMD (*F* (2,80) = 3.73, *p* < 0.05, η^2^ = 0.26). A post hoc Tuckey test showed that the non-adherent and moderate adherent groups of patients significantly differ on the level of experienced anxiety; the high adherence group was not significantly different from other two groups. Results indicate that the non-adherent group generally experiences more symptoms of anxiety than the moderate adherent group.

**Conclusions:**

Patients with major depressive disorder show significant non-adherence to medical treatment. More research is needed in this direction, as well as the development of recommendations and strategies to improve the level of adherence in this group of patients.

**Disclosure of Interest:**

None Declared

